# Modeling circadian and sleep-homeostatic effects on short-term interval timing

**DOI:** 10.3389/fnint.2015.00015

**Published:** 2015-02-17

**Authors:** Jakub Späti, Sayaka Aritake, Andrea H. Meyer, Shingo Kitamura, Akiko Hida, Shigekazu Higuchi, Yoshiya Moriguchi, Kazuo Mishima

**Affiliations:** ^1^Department of Psychophysiology, National Center of Neurology and Psychiatry, National Institute of Mental HealthTokyo, Japan; ^2^Division of Clinical Psychology and Epidemiology, Department of Psychology, University of BaselBasel, Switzerland

**Keywords:** sleep, circadian rhythm, time perception, interval timing, mixed models

## Abstract

Short-term interval timing i.e., perception and action relating to durations in the seconds range, has been suggested to display time-of-day as well as wake dependent fluctuations due to circadian and sleep-homeostatic changes to the rate at which an underlying pacemaker emits pulses; pertinent human data being relatively sparse and lacking in consistency however, the phenomenon remains elusive and its mechanism poorly understood. To better characterize the putative circadian and sleep-homeostatic effects on interval timing and to assess the ability of a pacemaker-based mechanism to account for the data, we measured timing performance in eighteen young healthy male subjects across two epochs of sustained wakefulness of 38.67 h each, conducted prior to (under entrained conditions) and following (under free-running conditions) a 28 h sleep-wake schedule, using the methods of duration estimation and duration production on target intervals of 10 and 40 s. Our findings of opposing oscillatory time courses across both epochs of sustained wakefulness that combine with increasing and, respectively, decreasing, saturating exponential change for the tasks of estimation and production are consistent with the hypothesis that a pacemaker emitting pulses at a rate controlled by the circadian oscillator and increasing with time awake determines human short-term interval timing; the duration-specificity of this pattern is interpreted as reflecting challenges to maintaining stable attention to the task that progressively increase with stimulus magnitude and thereby moderate the effects of pacemaker-rate changes on overt behavior.

## 1. Introduction

Interactions between the systems underlying temporal adaptation to the geophysical cycles of the environment (e.g., by generating near-24 h rhythms in physiology and behavior) and those mediating perception and action in relation to durations in the seconds-to-minutes range (i.e., short-term interval timing), have attracted research efforts on multiple levels, involving both animal and human subjects. Whereas animal research has made considerable progress in elucidating the neurobiological correlates of these interactions and some advances on this level have been made in human research (Shurtleff et al., 1990; Soshi et al., 2010; Agostino et al., 2011a,b; Bussi et al., 2014; Golombek et al., 2014), detailed quantitative characterization on the behavioral level and interpretation with reference to relatively abstract information-processing models of the functional relationships between these timing systems remain among the central aims in the pertaining human literature.

The referenced information-processing models generally remain neutral with respect to the concrete physiological implementation of the proposed mechanisms (but see e.g., Meck, 2003; Buhusi and Meck, 2005; Wittmann, 2013 for promising efforts at integrating explanatory levels), but they are mathematically and computationally tractable and generate specific predictions that can be assessed for consistency with behavioral data. These models thus continue to present powerful explanatory and predictive devices that, beyond their practical use, provide important guidance to experimentation and theorizing (Block, 1990; Lewandowsky and Farrell, 2010).

As a consequence, a model of this type, the so-called internal clock, or pacemaker-accumulator, model of interval timing, currently dominates human research into time-of-day and wake-dependent fluctuations in short-term interval timing (Pati and Gupta, 1994; Aschoff, 1998; Nakajima et al., 1998; Miro et al., 2003; Kuriyama et al., 2005; Moore and Gunzelmann, 2013) and, while by no means the only reasonable candidate, this model has seen very good success in accounting for a broad range of timing phenomena and, in terms of general functional principles, subsumes a number of physiologically inspired mechanisms; as such, the mechanism proposed by the pacemaker-accumulator model will probably continue to be of pragmatic use into the foreseeable future.

With respect to the suggested chronobiological effects on short-term interval timing, the proposition, typically, is, that the states of the circadian oscillator and the sleep homeostat (Borbely and Achermann, 1999) directly control the rate at which this mechanism's (Treisman, 1963) core pacemaker component emits pulses i.e., that this rate oscillates at a 24 h periodicity around an average defined by the exponential buildup and dissipation of the sleep homeostat's state across wakefulness and sleep, respectively (Pati and Gupta, 1994; Aschoff, 1998; Nakajima et al., 1998; Miro et al., 2003; Kuriyama et al., 2005; Moore and Gunzelmann, 2013).

**Figure 1:**
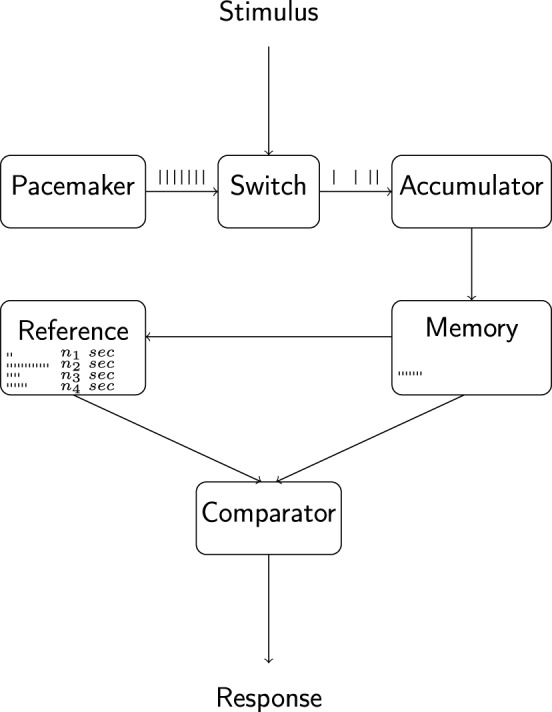
**Outline of the internal clock model of interval timing**.The pacemaker emits pulses at a rate that depends on organismic state and is hypothesized to be under the control of circadian and sleep-homeostatic processes. Pulses are gated into the accumulator and working memory components via an attention-controlled switch and attentional lability may lead to a leakage of pulses at this stage. The comparison of working memory contents with a reference memory containing pulse-duration associations formed during previous experiences constitutes the basis for overt behavior. Adapted from Wearden (unpublished manuscript).

The pulses emitted by the pacemaker, via an attention-controlled switch, reach a working memory module, or accumulator, where they are collected for comparison with the contents of a reference memory module containing pulse-duration associations acquired on previous occasions and attentional lability at the switch may introduce leakage of pulses from the system, causing a decrease in the effective number of pulses reaching the accumulator (i.e., a decrease in “effective” pacemaker-rate). A comparator component, given a criterion duration and on the basis of a continuous comparison of the contents of working memory with those of reference memory, elicits overt behavior as the number of pulses accumulated across a timing task reaches the number of pulses associated with the criterion (cf. Figure 1; for a detailed account of the historical development and variations of the model, see Wearden (unpublished manuscript). A multitude of a alternative models of interval timing of varied generality and level of implementation exist; for a review see e.g., Matell and Meck, 2004; for discussions regarding the putative neural substrates underlying interval timing see e.g., Meck, 2003; Matell and Meck, 2004; Buhusi and Meck, 2005; Coull et al., 2011; Wittmann, 2013).

In order to assess the plausibility of this proposed mode of interaction among timing systems, the model's predictions can be evaluated with respect to their compatibility with behavioral data; to this end, two well-established methods of human timing research lend themselves: the methods of duration estimation and duration production. In the estimation task, upon temporally delimited presentation of a stimulus, the experimental subject provides an estimate on that presentations' duration e.g., by entering a number (representing seconds of presentation) via a key-pad; conversely, in the production task, the experimental subject is presented a numeric representation of a target duration and responds e.g., by pushing a key after what he or she perceives to equal this duration.

The model, on the basis of the hypothesized circadian and sleep-homeostatic effects on pacemaker-rate, predicts specific performance changes across epochs of sustained wakefulness on each of these tasks, and a significantly improved fit (i.e., a greater improvement in fit than expected on the basis of increased model complexity alone) over the fit provided by an alternative model (such as the simple null hypothesis of no change across time) between the model's predictions and the observed data constitutes evidence in favor of the model (in analogy to the typical evaluation of e.g., simple linear regression or ANOVA models Estes, 1991; Maxwell and Delaney, 2003; Judd et al., 2008; Lewandowsky and Farrell, 2010).

For the *production* task, specifically, a relative increase in pacemaker-rate implies that the number of pulses corresponding (according to reference memory) to the requested duration is accumulated in a shorter amount of time and thus leads to a relative decrease in produced duration i.e., the relationship between pacemaker-rate and produced duration is reciprocal. Conversely, for the task of *estimation*, the number of pulses accumulated across the duration of stimulus presentation increases accordingly, and a relative increase in pacemaker-rate thus entails an increase in duration estimates. Analogous reasoning, naturally, extends to the case of a linear depression in pacemaker-rate, as well as to the more complex patterns of change in pacemaker-rate specified by the proposed circadian and sleep-homeostatic effects.

Accordingly, as a direct consequence of the model's design, a pacemaker-rate that oscillates at a 24 h periodicity (effect of the circadian oscillator) around an exponentially changing average (effect of changes to sleep-homeostat state), will entail a corresponding pattern in estimation performance and a reciprocal pattern in production performance, and this pattern, when expressed relative to stimulus duration (i.e., as the ratio *response*/stimulus), does not depend on stimulus magnitude.

If the assumption is made, however, that timing of shorter vs. longer durations differs in that maintaining stable attention to a stimulus becomes increasingly challenging with stimulus duration (Taatgen et al., 2007), the observed patterns for unequal stimulus magnitudes should diverge, leading to differing parameter values of the exponential and sinusoidal components in predicted behavior. More specifically, because, under this assumption, the number of pulses leaking from the system is assumed to be disproportionately greater for longer durations, the relative effect of a given increase in pacemaker-rate is smaller for the estimation of longer durations than it is for the estimation of shorter durations and thus leads to a widening gap across time between estimation trajectories for unequal durations; conversely, due to the *reciprocal* relationship between pacemaker-rate and *produced* duration, the model predicts a convergence across time of production levels for unequal durations.

Analogous reasoning naturally extends to the hypothesized compound exponential and sinusoid pattern characterizing the temporal development of pacemaker-rate and beyond that, circadian and sleep-homeostatic modulations to attentional lability could entail even more complex deviations from the identity pattern predicted if attention played no substantial role.

In reality, evaluation of the proposition, that circadian and sleep-homeostatic effects act in the above-specified manner on the mechanism to generate behavioral fluctuations, has proven rather difficult, as the pertinent human data is relatively sparse and lacking in consistency.

The reports on supportive evidence cited above are not free of methodological problems and are pitted against a number of studies relating negative or ambiguous results (Esposito et al., 2007; Späti, 2011; Pande et al., 2014), a situation that, according to some authors can, in part, be attributed to methodological and terminological incompatibilities across studies (Pande and Pati, 2010; Späti, 2011; Miguel, 2012; Moore and Gunzelmann, 2013). The prevalence of study designs that employ only singular timing methods and stimulus durations, the heterogeneity of instructions to subjects regarding the timing strategy to adopt and under-powered analytical approaches don't make optimal use of resources and further limit comparability of results.

As a consequence, we still lack clarity regarding the exact functional form of the suggested interaction and how it depends on task and stimulus duration, making evaluation of the proposed mechanism and the relative susceptibility of its components to circadian, sleep-homeostatic and, potentially, attentional, challenges difficult.

Here, in an effort to improve upon a number of these shortcomings, we aim at characterizing the conjectured chronobiological effects on interval timing by employing the methods of duration estimation and duration production on different target intervals within a unified experimental setting and, as a consequence, accumulate more reliable evidence regarding the proposed interaction between chronobiological and interval timing systems.

We chose to assess timing of two target durations via the tasks of duration estimation and duration production across two epochs of sustained wakefulness carried out within a constant routine setting under entrained (i.e., subjects' biological rhythms are synchronized with environmental cycles) and, respectively, free-running conditions (i.e., subject's biological rhythms are de-coupled from environmental cycles and run at their individual, intrinsic near-24 h, circadian, periodicities).

Following the theoretical considerations outlined above, we assumed both tasks to be reasonably characterized by a 24 h-oscillation around an average that follows a saturating exponential with time constant 18.2 h (Borbely and Achermann, 1999; Van Dongen et al., 2007, 2012) i.e., to follow a function of the form *Y*_*i*_ = *S*_*i*_ + *C*_*i*_ + ϵ_*i*_, where *i* is used to distinguish between individual subjects' curves across time i.e., between subjects' individual timing trajectories, *S* represents a saturating exponential component reflecting state of the sleep homeostat, *C* represents a sinusoid term reflecting state of the circadian oscillator and ϵ represents the deviations of the observed data points for subject *i* from the true subject-specific trajectory.

Trajectories are expected to vary in their parameters across subjects around averages defined by specific combinations of task, stimulus duration and constant routine i.e., by eventual significant simple and interactive effects of the factors under scrutiny and, following from the hypothesized endogenous character of the circadian and sleep-homeostatic effects, the pattern, when expressed relative to the endogenous rhythmicity in melatonin, is expected to remain stable across entrained vs. free-running conditions.

In summary, the theoretically motivated algebraic form chosen to characterize behavior across time in the individual can be compared with alternative forms (constant, sinusoid only, exponential only, sinusoid plus exponential, different vs. equal exponentials across durations, etc.) representing alternative hypotheses regarding simple and interactive effects of circadian phase, state of the sleep homeostat, etc. and, via embedding in an appropriately developed hierarchical structure, can readily accommodate the hypothesized effects of further factors and account for the expected variation in subjects' idiosyncratic trajectories.

## 2. Methods

**Figure 2:**
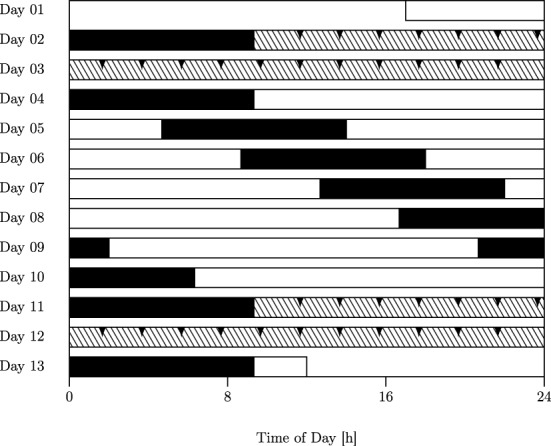
**Forced desynchrony (FD) protocol**. Filled bars, scheduled sleep (0 lx); open bars, wakefulness in dim light (15 lx); hatched bars, constant routine (CR). The FD protocol used in this study was a 28-h sleep–wake cycle consisting of alternating epochs of 9.33 h of sleep and 18.67 h of wakefulness. Interval timing tasks were administered at two-hourly intervals throughout both CRs (Days 2–3; Days 11–12; reported here; arrows indicate sampling points) as well as across the central part of the FD protocol (evening of Day 7 to afternoon of Day 8; not reported here).

Data on the production and estimation of 10 and 40 s was obtained from eighteen healthy young male subjects sampled at 2 h-intervals across two constant routine (CR) epochs conducted under conditions of sustained wakefulness (SD) of 38.67 h each, which were separated by an intervening 7 day-epoch of forced desynchrony (FD; cf. Figure 2).

### 2.1. Subjects

Eighteen young male subjects aged 19–39 years (mean ± sd: 22.44 ± 4.33 y) without any known sleep, physical, or psychiatric disorders or any history of using psychoactive drugs, as confirmed via a semi-structured interview conducted by a psychiatrist, all-night clinical polysomnography, blood chemistry tests, and several screening questionnaires, participated in the study; measures obtained from seventeen of these subjects have been previously published in entirely different contexts (Hida et al., 2013; Kitamura et al., 2013). None of the subjects had worked night shifts or traveled across time zones within 6 months preceding the study; none of the participants displayed clinical signs of visual impairment and fundus examination detected no morphologic abnormalities of the retina. The study design was approved by the local ethics committee and all participants gave their informed consent; all procedures conformed to the Declaration of Helsinki.

### 2.2. Protocol

Subjects underwent a 13-day protocol in a temporal isolation laboratory devoid of external time cues (previously described in Kitamura et al., 2013). Briefly, participants entered the laboratory at 5 P.M. on Day 1 and, after having a meal and taking a bath, turned the lights off and went to bed at 12 A.M. The protocol, which started the following morning after 9.33 h of bed rest, comprised measurement of melatonin rhythm and interval timing under constant routine conditions (CR1) followed by a 28 h sleep-wake schedule for 7 days, and a second measurement of melatonin rhythm and interval timing under constant routine conditions (CR2).

The intervening 28 h sleep-wake schedule consisted of alternating cycles of 9.33 h of scheduled sleep (promoting sleep/bed-rest with lights off) and 18.67 h of scheduled wakefulness (prohibiting sleep). Throughout the study, subjects were under constant surveillance by a researcher and were verbally awakened when they unintentionally fell asleep during the wake period. Subjects were asked to maintain a semi-recumbent posture under low-intensity light conditions (< 15 lx) and consume small meals (approx. 200 kcal) at 2-h intervals; water was the only source of liquid intake and was available *ad libitum* (no other beverages, including coffee or any other alertness-boosting beverages were allowed). During wake periods, participants were allowed to move freely around the laboratory, read and write, enjoy music and videos, play video-games, and engage in conversation with the researcher. During scheduled sleep, subjects were asked to sleep in the bedroom with lights off (0 lx).

### 2.3. Measures

#### 2.3.1. Melatonin

During each 38.67 h-epoch of sustained wakefulness, blood samples were collected at 60 min intervals via a stopcock attached directly to an intravenous catheter and centrifuged; the plasma collected was frozen at −80°C for radioimmunoassay of melatonin concentrations.

#### 2.3.2. Interval timing

In order to trace the relationship between objective and subjectively perceived duration across the study protocol, we used two methods classically employed in human interval timing research i.e., the methods of estimation and production (Clausen, 1950; Bindra and Waksberg, 1956; Wallace and Rabin, 1960), which were implemented using the E-Prime stimulus presentation software (version 1.1.4.6) on a laptop computer.

In the estimation task, upon temporally delimited presentation of a stimulus (filled red circle on white background, centered on the display of the laptop computer), the participant provided an estimate on that presentations' duration by entering a number (representing seconds of presentation) via a key-pad. In the production task, the participant was presented a numeric representation of a target duration (white number on blue background, centered on the display of the laptop computer and representing duration in seconds to produce) and pushed a key after what he perceived to equal this duration. In each interval timing session all subjects performed temporal estimation and production of 10 and 40 s. At each measurement occasion, each stimulus/task combination was given three times in randomized sequence, each measurement occasion lasting approx. 7 min.

### 2.4. Analysis

#### 2.4.1. Melatonin

Plasma melatonin concentrations were measured using a radioimmunoassay (RIA) technique (SRL, Tokyo, Japan) at an assay sensitivity of 2.8 pg/ml. DLMO (dim light melatonin onset) was defined as the time of a cosine-fitted curve, when plasma melatonin concentrations rose from a low background level to above 10 pg/ml (24/12-h composite cosine model fitted to the z-score standardized data using ChronoLab 3.0).

#### 2.4.2. Interval timing

Estimation and production data was analyzed using a random coefficient model in R's (version 3.1; R Core Team, 2014) nlme (Pinheiro et al., 2015) package.

The ratios of the subject's average response to the target (stimulus-) duration at each measurement occasion, multiplied by 100 for computational reasons, served as the source to further analyses. Values of this measure above one hundred thus denote over(estimation/production) and values of this ratio below one hundred denote under(estimation/production); a value of one hundred for this ratio implies exact, veridical, estimation/production. Outcome values were removed from the dataset prior to analysis if their standardized value was ≥ 250.

The following predictors were used as fixed factors: task (production, estimation; TASK), stimulus (10, 40 s; STIM), constant routine (CR1, CR2; CR) and time relative to DLMO.

In the model building process, we included the exponential function exp(−*t*/18.2), representing state of the sleep homeostat across sustained wakefulness (Borbely and Achermann, 1999; Van Dongen et al., 2007, 2012) as well the predictors sin(2π/24*t*) and cos(2π/24*t*), jointly representing state of the circadian oscillator (due to the equivalence *A* · cos(ω*t* − *P*) = *s* · sin(ω*t*) + *c* · cos(ω*t*)) for time.

Theoretically motivated interactive terms were included if they improved model fit, which was evaluated using likelihood ratio tests; we included a random intercept and random slopes for stimulus and task as this improved model fit; also, the model fitted different variances by tasks and stimuli due to variance heterogeneity among groups.

The model developed was:

$$\begin{array}{l} y_{ij}=\beta_{0}+\beta_{1}TASK_{ij}+\beta_{2}STIM_{ij}+\beta_{3}CR_{ij}\\ \;+\beta_{4}\exp(-t_{ij}/18.2)+\beta_{5}\sin(2\pi /24t_{ij})+\beta_{6}\cos(2\pi /24t_{ij})\\ \;+\beta_{7}TASK_{ij}\times STIM_{ij}+\beta_{8}TASK_{ij}\times CR_{ij}\\ \;+\beta_{9}TASK_{ij}\times \exp(-t_{ij}/18.2)+\beta_{10}TASK_{ij}\times \sin(2\pi /24t_{ij})\\ \;+\beta_{11}TASK_{ij}\times \cos(2\pi /24t_{ij})+\beta_{12}STIM_{ij}\times \exp(-t_{ij}/18.2)\\ \;+\beta_{13}STIM_{ij}\times \sin(2\pi /24t_{ij})+\beta_{14}STIM_{ij}\times \cos(2\pi /24t_{ij})\\ \;+b_{0i}+b_{1i}TASK_{ij}+b_{2i}STIM_{ij}+\epsilon_{ij} \end{array}$$

where *i* = subject, *j* = time point, *y*_*ij*_ = response (produced, estimated duration), *STIM*_*ij*_ = stimulus duration (10, 40 s), *CR*_*ij*_ = constant routine (CR1, CR2), *TASK*_*ij*_ = task (estimation, production), *t*_*ij*_ = time from DLMO in hours, β_1_ to β_14_ = regression coefficients of the independent variables, *b*_0*i*_ = subject-specific random intercept and, *b*_1*i*_ and *b*_2i_ = subject-specific random slopes for task and stimulus, respectively.

## 3. Findings

Consistent with our hypothesis of circadian and sleep-homeostatic control of an internal clock model's pacemaker rate, estimation and production of two target durations is readily accommodated by a random coefficient-model for both tasks that comprises a saturating exponential term as well as simple trigonometric terms relating duration production and estimation to states of the sleep homeostat and the circadian oscillator.

**Figure 3:**
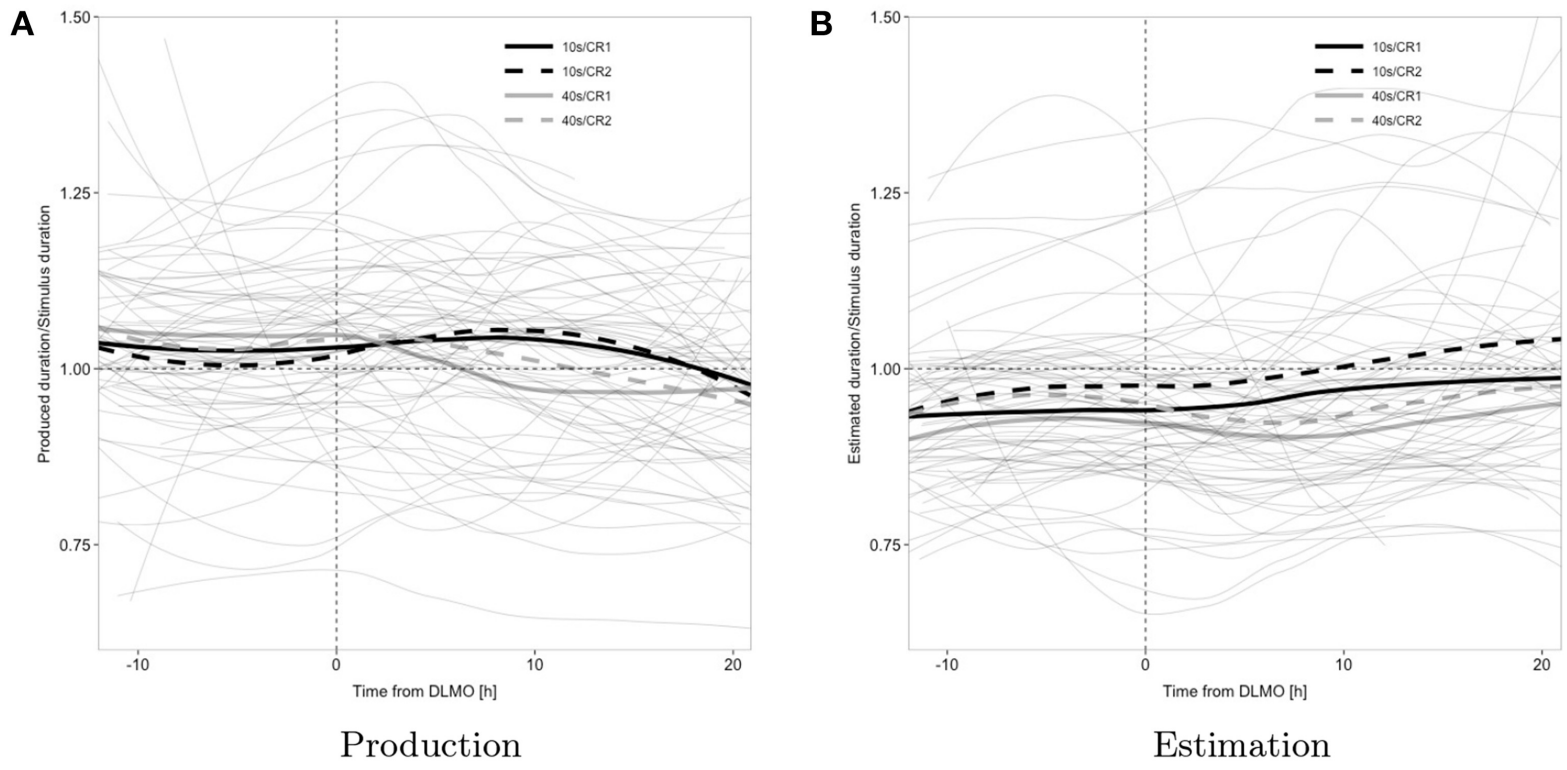
**Observed data; individual subject's loess smoothed trajectories (thin solid gray lines; one trajectory per subject and condition) and average time course across all subjects by target duration and constant routine (thick; black, 10 s; gray, 40 s; solid, CR1; dashed, CR2); the horizontal reference line represents veridical timing; the vertical reference line represents dim light melatonin onset (DLMO)**.

Trajectories of duration estimation and production, during each epoch of sustained wakefulness, displayed a relatively large degree of inter-individual variability, both in terms of unconditional levels and in terms of effects of task and target duration on these levels, as indicated by the fact that inclusion of a random intercept and random slopes for stimulus and task significantly improved model fit (cf. Figure 3), which may suggest subject-specific variation in baseline arousal and attentional level as well as potentially differing timing strategies.

**Figure 4:**
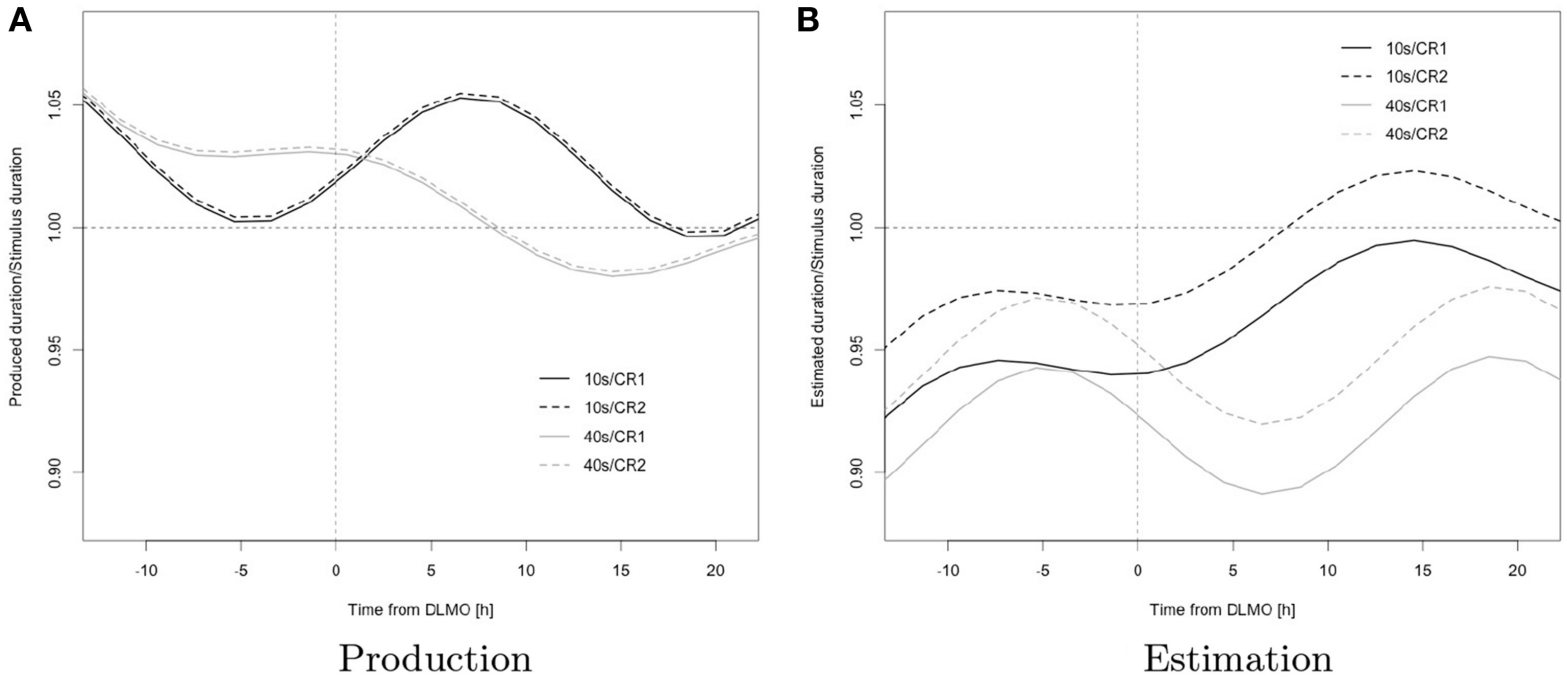
**Model predictions; black, 10 s; gray, 40 s; solid, CR1; dashed, CR2; the horizontal reference line represents veridical timing; the vertical reference line represents dim light melatonin onset (DLMO)**.

The sinusoidal component of the response to target-ratio's trajectory (main effect sin(2π/24*t*)), on average, varied across the two tasks of estimation and production (interaction sin(2π/24*t*) × *TASK*) as well as across the target intervals of 10 and 40 s (interactions *STIM* × sin(2π/24*t*) and *STIM* × cos(2π/24*t*)); the exponential component of the response-to-target ratio's trajectory, across subjects and averaged across the tasks of estimation and production, was more positive for the 10 s stimulus (overall trend positive), than for the 40 s stimulus (overall trend negative; interaction *STIM* × exp(−*t*/18.2)). The ratio was typically below one for estimation (underestimation) but increased over time to reach values close to one, whereas it began above one for production (overproduction) to decrease over time and finally reach values below one (under-production; interaction exp(−*t*/18.2) × *TASK*) (cf. Table 1 and Figure 4).

**Table 1:** **Estimated coefficient by model term**.

**Effect**	**Value**	**Std.Error**	**DF**	***t*-value**	***p*-value**
(*Intercept*)	102.13	2.32	2424	43.95	0.00
*CR*	0.19	0.79	2424	0.23	0.81
*STIM*	−4.72	1.72	2424	−2.75	0.01
exp(−*t*/18.2)	2.24	3.23	2424	0.69	0.49
sin(2π/24*t*)	2.64	0.68	2424	3.86	0.00
cos(2π/24*t*)	−0.92	0.68	2424	−1.36	0.17
*TASK*	−2.38	4.83	2424	−0.49	0.62
*CR* × *TASK*	2.66	1.06	2424	2.51	0.01
*STIM* × *TASK*	−2.84	1.08	2424	−2.62	0.01
exp(−*t*/18.2) × *TASK*	−17.19	3.56	2424	−4.83	0.00
sin(2π/24*t*) × *TASK*	−3.28	0.75	2424	−4.38	0.00
cos(2π/24*t*) × *TASK*	−0.63	0.74	2424	−0.86	0.39
*STIM* × exp(−*t*/18.2)	13.29	3.58	2424	3.71	0.00
*STIM* × sin(2π/24*t*)	−2.05	0.76	2424	−2.69	0.01
*STIM* × cos(2π/24*t*)	2.20	0.75	2424	2.94	0.00

This pattern is consistent with a wake-dependent increase in addition to a circadian oscillation in the rate at which the underlying pacemaker emits pulses as this, as outlined in the introduction, entails a corresponding pattern in estimation and a reciprocal pattern in production.

The variation in the expression of the pattern in function of stimulus duration further suggests an interaction of change in pacemaker rate with attentional factors in determining overt behavior: specifically, the more positive exponential component for the trajectory of 10 s, when compared to that for 40 s (reflected in a steeper increase in estimation and shallower decrease in production associated with this duration), suggests that duration-specific attentional demands moderate the effects of changes in pacemaker-rate on overt timing behavior (*STIM* × exp(-*t*/18.2)), thus leading to unequal “effective” pacemaker rates (i.e., the actual rates at which pulses reach the accumulator) across durations; the observed pattern is consistent with a pulse loss at the attention-gated switch that progressively increases with target duration and may reflect an increase in attentional lability with target duration (Taatgen et al., 2007). As a consequence, a given increase in pacemaker rate has relatively less effect on the increase in estimation for longer durations than it does for shorter durations, leading to a widening gap between the exponential components in estimation. Due to the reciprocal relationship between pacemaker rate and production, we observe a convergence for production, and analogous reasoning extends to duration-specific characteristic of the sinusoidal terms.

This interpretation is further supported by the observation of unequal amplitudes in the oscillatory component for short vs. long durations (*STIM* × sin(2π/24*t*)); the observed phase difference in oscillation across durations (*STIM* × cos(2π/24*t*)) may however suggest additional circadian and/or sleep-homeostatic effects on attention (over and above the respective effects on pacemaker-rate).

The ratio was higher for the 10 s than for the 40 s stimulus and during CR2 vs. CR1 in estimation, whereas no corresponding differences were observed in production (interactions *STIM* × *TASK*, *CR* × *TASK*) a finding that, again, may be accounted for by the fact that the internal clock model directly relates estimation to pacemaker-rate but specifies a reciprocal relationship between pacemaker-rate and production and thus leads to different behavioral consequences of changes in pacemaker-rate across tasks (*STIM* × *TASK*).

Again, as outlined above, an increasing pacemaker rate, combined with differential pulse loss due to attentional fluctuations across durations, leads to different rates of increase in the response across durations, thus accounting for the widening gap evident in estimation ratios across different durations; conversely produced duration-ratios across targets progressively converge.

In combination with a hypothesized greater attentional stability during the second constant routine, which may be attributed to the effect of habituation to the task, the unequal relationship between estimated and produced durations to pacemaker-rate may also account for the significant increase in level across constant routines observed for estimation that is absent from production (*CR* × *TASK*): a relative increase in “effective” pacemaker-rate across constant routines, due to the direct relationship, is bound to affect overall levels of the response more strongly for the task of estimation and thus lead to the observed difference in effects across the tasks of estimation and production.

## 4. Discussion and outlook

Our observations and interpretation are generally in line with the results reported in Späti (2011) but reveal the more appropriate design of our study which allows for testing of specific hypotheses, including the more adequate nature of a random coefficient modeling approach to evaluation of this kind of data: the repeated measures analysis of variance approach to assessing production and reproduction trajectories collected under sustained wakefulness used in Späti (2011) identified the dependence of trajectories on task and stimulus magnitude but, possibly due to a lack in statistical power, failed to determine the circadian and wake-dependent modulations in production suggested by visual inspection; here, on the other hand, using a random coefficient-modeling approach to the analysis of chronobiological time series, we were able to suggest and test the exact way in which interval timing trajectories are affected by the interaction of circadian and homeostatic effects with stimulus magnitude and nature of the task and, as a consequence, test more specific hypotheses (for recent, accessible overviews on some of the shortcomings of more traditional approaches to the analysis of longitudinal data and how these are addressed by random coefficient modeling, see Winter, 2013; Finch et al., 2014; Mirman, 2014 as well as the more thorough treatments in Singer and Willett, 2003, Pinheiro and Bates, 2009 and Long, 2011; for application to a chronobiological context in R and, respectively, SAS, see Seltman, 1997; Albert and Hunsberger, 2005; for further examples on the use of R in a chronobiological context, see Barnett and Dobson, 2010, as well as Lee Gierke et al., 2013, Qiu et al., 2014 and Sachs, 2014).

Further comparison with the pertinent literature, corroborate a picture drawn by our data that is largely consistent with previous findings but of much greater detail and thus more theoretically informative: Nakajima et al., in a 36 h-sleep deprivation study on four healthy young men involving production of 10 and 60 s, observed an oscillatory pattern in responses with minimal production of 10 s around 10 P.M. and minimal production of 60 s around 7 P.M., a pattern that is consistent with the general trends observed for production, for our data (Nakajima et al., 1998). Also in line with our findings are results reported by Kuriyama et al. who, during 30 h of sustained wakefulness involving 8 subjects, reported an oscillatory pattern with a minimum in the evening (17–21 h) for the production of 10 s (Kuriyama et al., 2005).

As was the case for the study reported here, Kuriyama et al. explicitly discouraged subjects from counting and rhythmisizing; as it remains unclear however, what instructions were given to subjects on this issue in the study reported by Nakajima et al., comparisons with this experiment need to be understood with caution.

In the studies mentioned below, on the other hand, subjects were either instructed to count and rhythmisize or the design employed was very different from the one used in our study in a number of other aspects; these studies are thus reported here primarily to illustrate the heterogeneity of methodologies and findings and, as a consequence, comparisons with the data reported here are rather difficult to draw.

Soshi et al., after one night of sustained wakefulness involving 18 subjects in a pre/post comparison (21:00–09:00), found a decrease in the production of 10 s (but an increase under conditions of sleep satiation) (Soshi et al., 2010) and Aschoff, in production of 5 and 10 s by seven subjects, reported an anti-cyclical oscillation to core body temperature but no association with wake time (Aschoff, 1998).

In contrast, Pati and Gupta in 10 subjects, under everyday conditions and employing a counting strategy, reported a parallel oscillation of 10 s production with core body temperature (Pati and Gupta, 1994) and Esposito et al., using a 15-s rhythmisizing task on 54 subjects across one night of sustained wakefulness found neither circadian nor sleep-homeostatic effects (Esposito et al., 2007).

On the other hand, Miro et al., using 10 s production under a counting paradigm across 60 h of sustained wakefulness, reported circadian minima occurring around 17–21 h that combined with a linearly increasing component across the protocol (Miro et al., 2003).

This heterogeneity in findings and the contrast with our results, again, stresses the importance of careful distinction between designs. A limitation with our study shared with those carried out by other groups is the relatively poor control on the strategies adopted by individual subjects. In some of the aforementioned studies, the authors encouraged counting or sequencing strategies, while other reports give no information about the exact instructions given and/or compliance with them. In our study, counting or sequencing were explicitly discouraged but compliance is difficult to control and, possibly, also difficult to expect in the tasks of production and estimation, where durations are actually given/requested numerically. A simple suggestion for improvement that circumvents the introduction of distractor tasks, is the use of *post-hoc* questionnaires to be completed after each timing session which provide the participants with a means to self-evaluate their performance with respect to the strategies employed and that can be accounted for in the data analytic process.

In summary, while generally in line with previous reports on the circadian and sleep-homeostatic modulation of interval timing, our results, by combining assessment of production and estimation of two stimulus magnitudes across sustained wakefulness under entrained and free-running conditions with the more powerful approach of random coefficient modeling to data analysis, draw a much more detailed picture and allow for more specific and robust inferences regarding the putative operation of the hypothesized pacemaker-accumulator mechanism, the adequacy of which in accounting for critical features of the data we could confirm and, concurrently refine: whereas our findings are consistent with the hypothesis of circadian and sleep-homeostatic modulations in the rate of an underlying pacemaker and suggest that its pulse rate oscillates at a 24 h-period around an increasing saturating exponential with time constant 18.2 h, our results also point at an important role of attentional demands in timing a given duration, by moderating the impact of pacemaker rate on observed behavior; changes in pacemaker rate alone cannot explain the full complexity of the observed pattern.

Further evaluation of interactions between the timing systems studied in chronobiology one the one hand and cognitive psychology and psychophysics on the other hand should profit from an extension of our approach encompassing tasks that avoid translation to and from explicit numerical representations, from careful control on subjects' strategies as well from the use of different target intervals in order to assess the limits to which the model can accommodate the data. Chronobiological interventions in concert with measures aimed at further separating the relative contribution of attentional factors from those related to pacemaker rate, such as concurrent assessment of physiological and psychological parameters, should support further theorizing. Future research should be directed at whether more physiologically informed models can account for the data, as well as at what role e.g., modulation in dopaminergic pathways (Buhusi and Meck, 2005; Bussi et al., 2014; Wearden, unpublished manuscript) may play in accounting for the behavior observed (dopaminergic transmission has been shown to be relevant in the circadian modulation of interval timing and to be associated with pacemaker-rate in the internal clock model as well as with frequency and synchronization of cortical oscillations and resetting of the membrane properties of striatal spiny neurons on stimulus onset in the physiologically informed striatal beat frequency model Matell and Meck, 2004; Buhusi and Meck, 2005; Meck et al., 2008). Finally, concurrent measurement of higher-order timing faculties may provide valuable insights into human timing beyond the relatively narrow limits studied here and help elucidate as of yet poorly understood phenomena such as cognitive temporal orientation (Späti et al., 2009) (but see Wackermann, 2014 for a discussion of caveats regarding unreflected generalization of concepts across domains).

### Conflict of interest statement

The authors declare that the research was conducted in the absence of any commercial or financial relationships that could be construed as a potential conflict of interest.
